# Occupational Health: Agriculture–COPD Link Bolstered

**DOI:** 10.1289/ehp.115-a444

**Published:** 2007-09

**Authors:** Bob Weinhold

Smoking is typically considered the cause of 80–90% of chronic obstructive pulmonary disease (COPD) cases, but another significant known source is occupational exposure. Among the suspect occupations is farming, for which there has long been limited evidence of a risk for various respiratory diseases, including COPD. That link has been bolstered by an Austrian study, based on clinical evidence, published in the June 2007 issue of the *American Journal of Industrial Medicine*.

“This is really the first study that shows a clear association confirming the agricultural risk,” says Alfred Munzer, director of pulmonary medicine at Washington Adventist Hospital in Takoma Park, Maryland, who did not participate in the study. Other studies have generally been much smaller or observational, he adds.

Lead author Bernd Lamprecht, a pulmonologist at Paracelsus Medical University in Salzburg, says this is the first population-based study to evaluate COPD on the basis of measured postbronchodilator lung function, in combination with questionnaire data on occupational exposure. In the study, the team evaluated 1,258 Salzburg-area residents aged 40 and older, and found that 23% had any type of farming experience (farms in the area tend to be small, with a mix of various crops and animals). Substantially fewer farmers (46.2%) than non-farmers (54.6%) were current or former smokers. But 30% of the farmers had at least mild COPD, as determined by spirometer measurements of lung function before and after inhalation of a bronchodilator drug, compared with 22% of the nonfarmers. Using criteria for more severe COPD, the relative difference was even greater, with 14% of farmers and 8% of nonfarmers impaired. For mild COPD, the differences began showing up at about age 50, and held beyond age 80; corresponding data for more severe COPD were not reported.

The authors acknowledge numerous limitations of their study, such as the lack of information on the type, duration, and intensity of exposures for the farmers, the limited range in geography and farm type, and the lack of long-term spirometry data for the study subjects. They could not speculate on what exposures may have caused the elevated COPD, but note that other studies have linked agricultural dusts with various respiratory problems.

Another important consideration is that the method of classifying COPD used in the study is relatively new and has not been validated against the methods historically used to apply a COPD diagnosis, says Neil Schachter, a professor of medicine in pulmonary and critical care at Mount Sinai Medical Center. The new criteria were developed by the WHO and the National Heart, Lung, and Blood Institute through their Global Initiative for Chronic Obstructive Lung Disease (GOLD) and were released in 2001. The criteria are designed to provide more rigorous guidelines for a COPD diagnosis, putting substantial emphasis on spirometry results and relying less on patient history, symptoms, other tests, and a doctor’s judgment, as has been the case to date.

Schachter points out that 5% of all the study subjects, both farmer and nonfarmer, reported having a doctor’s diagnosis of COPD, which is far lower than the 30% and 22% numbers reported by Lamprecht’s team. This finding highlights the long-running controversy over how to diagnose COPD, and what specific disease labels—such as emphysema, chronic bronchitis, some forms of asthma, and hypersensitivity pneumonitis—should be included under the COPD umbrella.

Nonetheless, with COPD the fourth leading killer worldwide, identifying the causes of this disease is an important task. The limitations of the Austrian study need to be addressed, but Schachter says, “I think they found a real phenomenon: farmers are a high-risk category.”

## Figures and Tables

**Figure:**
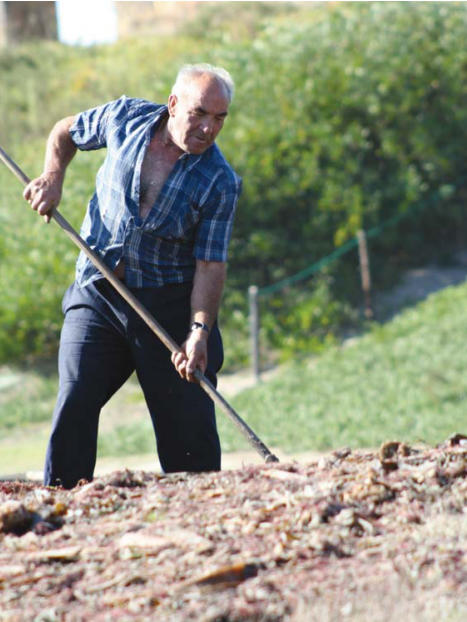
Soil risk? Exposure to mineral dusts stirred up by working the soil could contribute to COPD, a progressive disease characterized by limited airflow, chronic cough, and increased bronchial mucus secretion.

